# Molecular epidemiology and hematological profiles of hemoglobin variants in southern Thailand

**DOI:** 10.1038/s41598-024-59987-4

**Published:** 2024-04-22

**Authors:** Wanicha Tepakhan, Sataron Kanjanaopas, Korntip Sreworadechpisal, Tipparat Penglong, Pornpun Sripornsawan, Chaowanee Wangchauy, Chadaporn Nokkong, Chulalak Kongkan, Saristha Buathong

**Affiliations:** 1 Department of Pathology, Faculty of Medicine, Prince of Songkla University, Songkhla, 90110 Thailand; 2 Department of Pediatrics, Faculty of Medicine, Prince of Songkla University, Songkhla, 90110 Thailand

**Keywords:** Molecular biology, Molecular medicine, Genotype, Medical genetics, Mutation, Epidemiology, Genetics research

## Abstract

Data on hemoglobin (Hb) variants in southern Thailand are lacking. This study aimed to reassess the frequency of Hb variants and the clinical aspects of compound heterozygous Hb variant with other hemoglobinopathies. We enrolled 13,391 participants from ten provinces in southern Thailand during 2015–2022. Hb analysis was performed using capillary electrophoresis, and mutations in the *HBA* and *HBB* genes were identified using PCR or DNA sequencing. Hb variants were identified in 337 (2.5%) unrelated subjects. Nine β-chain variants, namely Hb Malay (76.9%), Hb C (10.1%), Hb D-Punjab (2.9%), Hb G-Makassar (2.3%), Hb Dhonburi (2.3%), Hb Tak (1.4%), Hb J-Bangkok (1.4%), Hb New York (0.3%), and Hb Hope (0.3%), and four α-chain variants—Hb G-Georgia (*HBA1*) (0.9%), Hb G-Georgia (*HBA2*) (0.3%), Hb Q-Thailand (0.6%), and Hb St. Luke’s-Thailand (0.3%)—were identified. The southern population exhibited a distinct spectrum of Hb variants compared to that observed in the populations from other areas. Several compound heterozygous genotypes were also identified. Combining Hb Malay with Hb E or high Hb F determinants did not require a blood transfusion. This study provides essential information for genetic counseling in thalassemia prevention and control programs in this region.

## Introduction

Hemoglobin (Hb) variants, or abnormal Hb, are hemoglobinopathies resulting from an abnormal structure of the globin chain in the hemoglobin molecule. Several Hb variants, such as Hb E (*HBB*:c.79G>A) and Hb Malay (*HBB*:c.59A>G), termed as “thalassemic Hb variants” can lead to reduced Hb variant levels. In Thailand, over 30 types of Hb variants have been reported, with a prevalence rate of 2.4%. However, Hb variants exhibit variations across populations and countries^1^. The interaction between Hb variants and thalassemia typically manifests as either no or mild clinical phenotypes^2,3^. Nevertheless, this co-inheritance may result in the misinterpretation of Hb analysis within thalassemia prevention and control programs. For example, certain Hb variants co-migrate within the Hb F zone of capillary electrophoresis (CE), leading to potential misdiagnosis as β-thalassemia disease or high Hb F determinants until molecular diagnosis confirms the specific mutation type^4^. In addition, the interaction of thalassemic Hb variants with thalassemia can contribute to moderate to severe thalassemia phenotypes, as seen in Hb H with Hb Constant Spring (CS, *HBA2*:c.427T>C) disease and Hb E/β-thalassemia disease, which are commonly observed in the southeast Asian population^5,6^. Eight Hb variants in 58 southern populations were previously identified using high-performance liquid chromatography (HPLC) and DNA sequencing^7^. However, the report did not include Hb Malay, a common Hb variant in southern Thailand and lacked hematological profiles of the combination of Hb Malay with other Hb variants. Up-to-date CE technique is an Hb analysis routinely performed in most laboratories in Thailand. Our center started using this method in 2015. Moreover, there is limited information on the spectrum, prevalence, and clinical phenotypes of Hb variants when co-inherited with other abnormalities in southern Thailand using the CE method. Therefore, this study aimed to reassess the molecular epidemiology of Hb variants and the clinical phenotypes of patients with co-inherited Hb variants and other thalassemia or hemoglobinopathies in the southern population.

## Results

### Genotypic and phenotypic spectra of Hb variants in the southern Thai population

**Figure 1:**
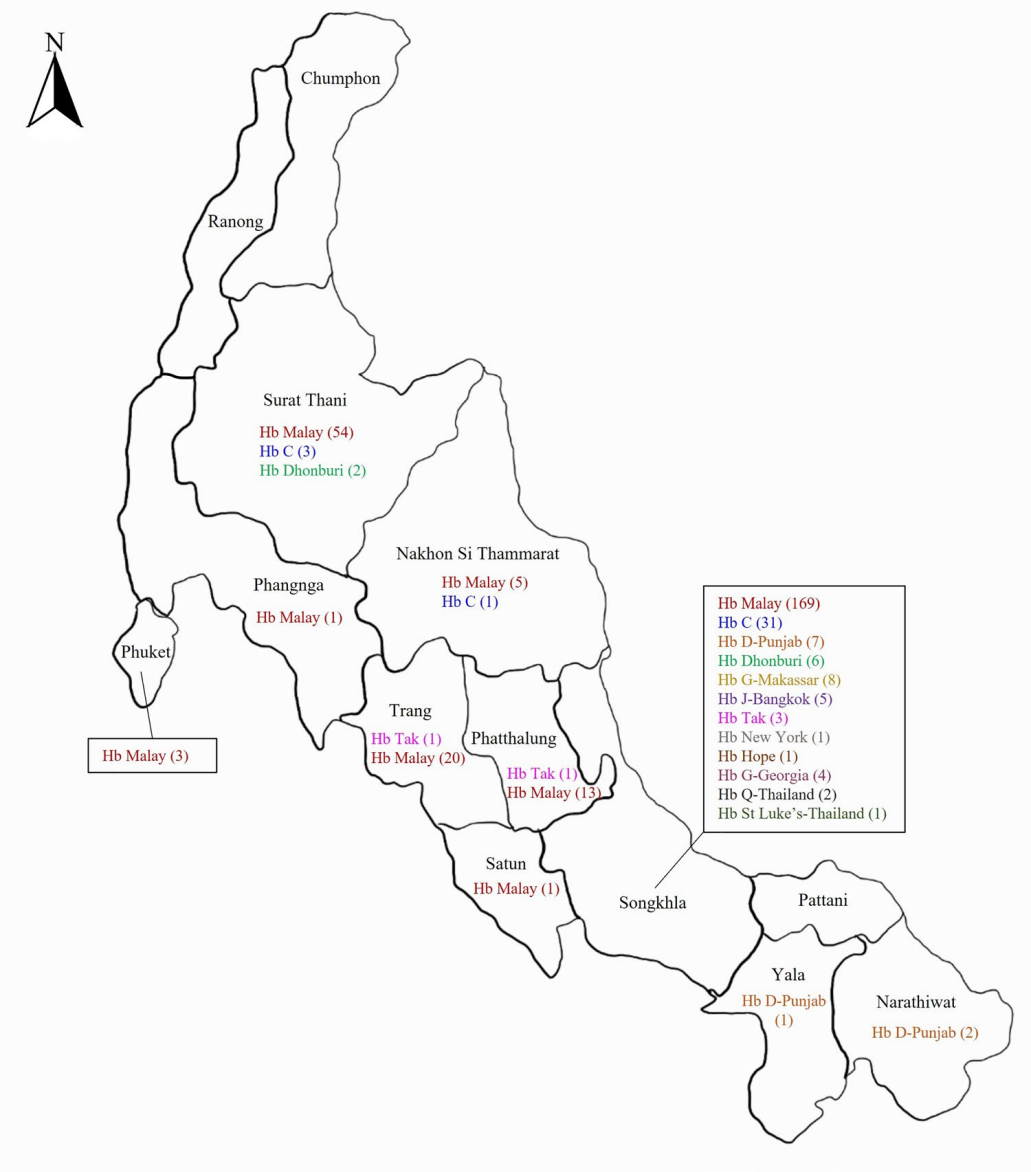
Distribution of Hb variants among 346 chromosomes of 337 participants across ten provinces of southern Thailand. The number represents the allelic count of Hb variants.

**Figure 2:**
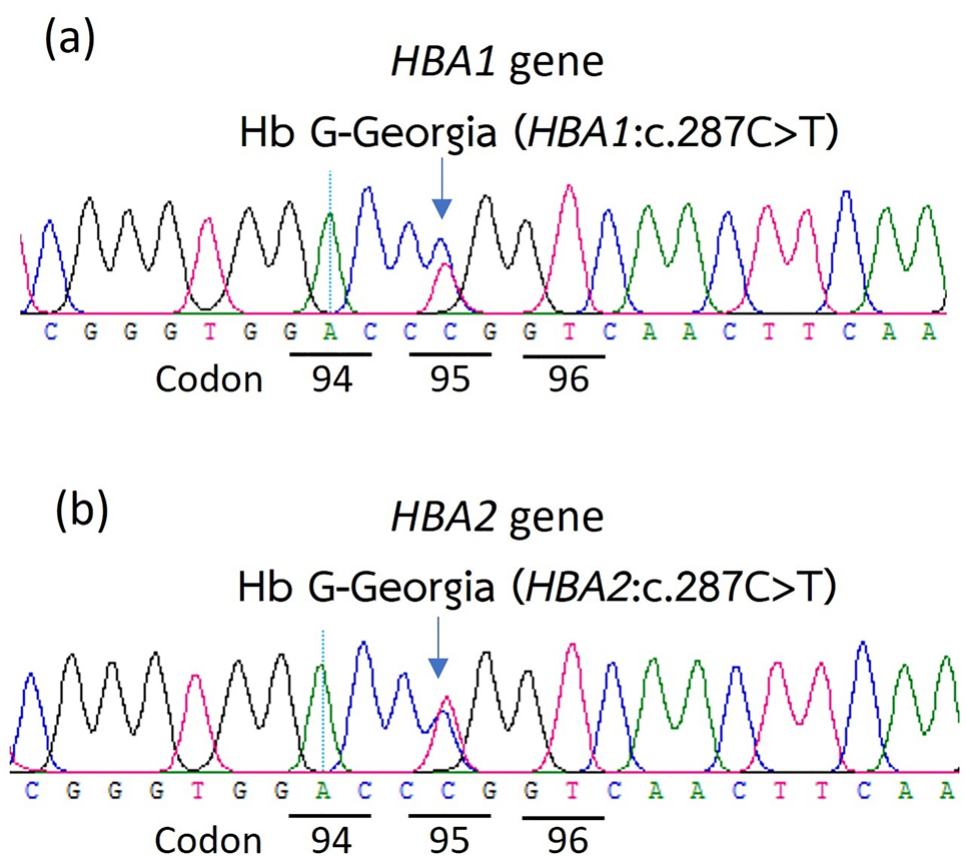
Sanger DNA sequencing results for Hb G-Georgia (*HBA1*) and (*HBA2*).

**Figure 3:**
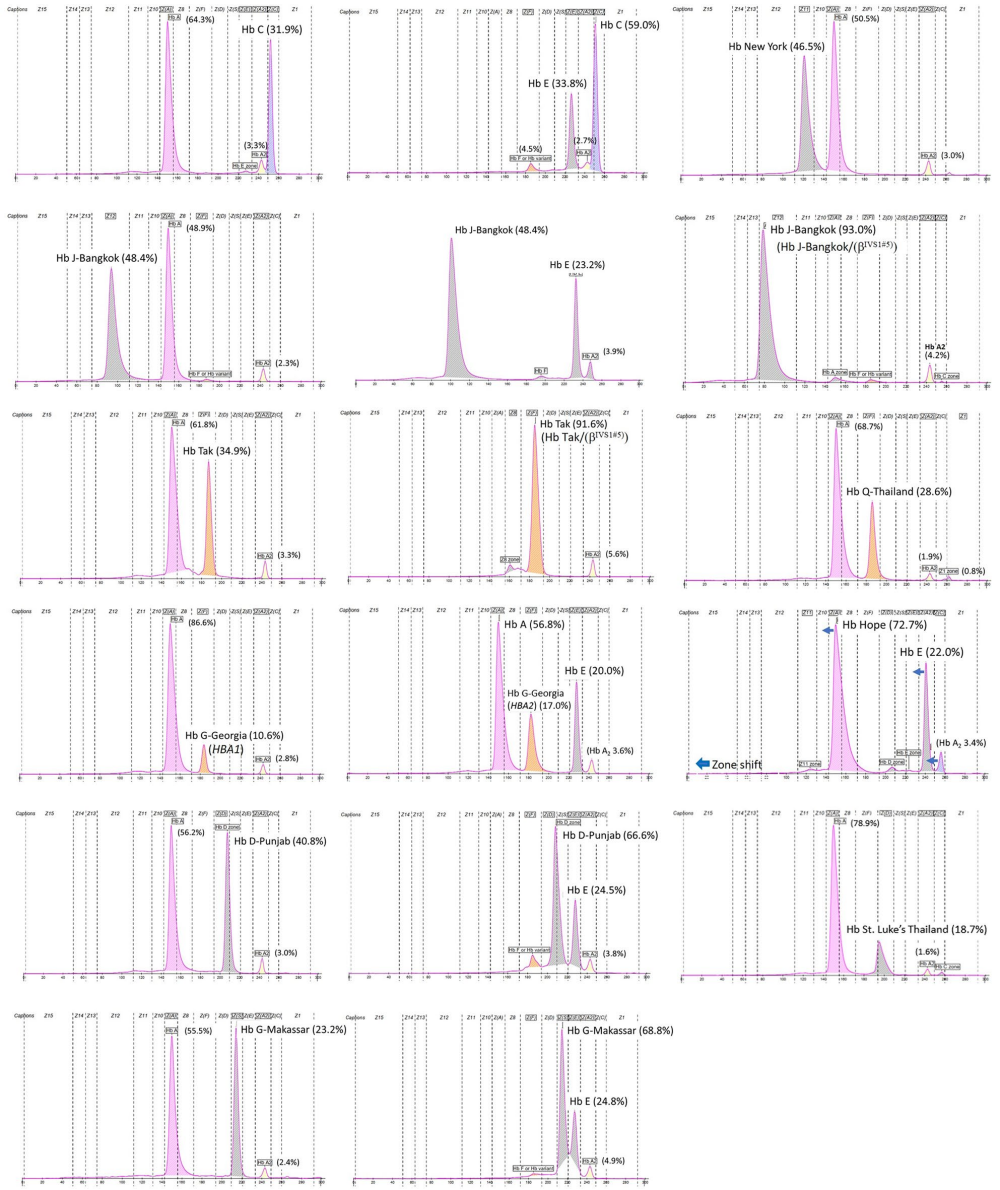
Hemoglobin (Hb) analysis results of Hb variants from southern populations using the capillary electrophoresis method.

Our reference center received 13,391 samples for thalassemia and hemoglobinopathy diagnoses from 10 provinces in southern Thailand, namely Songkhla, Surat Thani, Nakhon Si Thammarat, Phatthalung, Trang, Phuket, Phangnga, Satun, Yala, and Narathiwat, spanning the period from 2015 to 2022. Routine molecular diagnosis has revealed Hb Malay and Hb Dhonburi mutations and DNA sequencing has revealed 11 distinct Hb variant mutations in 337 (2.5%) unrelated participants, corresponding to 346 chromosomes. The β-chain variant, with nine different mutations, was identified in 338 alleles, and the two most common mutations were Hb Malay (n = 266, 76.9%) and Hb C (*HBB*:c.19G>A) (n = 35, 10.1%). The remaining seven mutations were Hb D-Punjab (*HBB*:c.364G>C) (n = 10, 2.9%), Hb G-Makassar (*HBB*:c.20A>C) (n = 8, 2.3%), Hb Dhonburi (*HBB*:c.380T>G) (n = 8, 2.3%), Hb Tak (*HBB*:c.441_442insAC) (n = 5, 1.4%), Hb J-Bangkok (*HBB*:c.170G>A) (n = 5, 1.4%), Hb New York (*HBB*:c.341T>A) (n = 1, 0.3%), and Hb Hope (*HBB*:c.410G>A) (n = 1, 0.3%). In addition, four α-chain variants, namely Hb G-Georgia (*HBA1*) (*HBA1*:c.287C>T) (n = 3, 0.9%), Hb G-Georgia (*HBA2*) (*HBA2*:c.287C>T) (n = 1, 0.3%), Hb Q-Thailand (*HBA1*:c.223G>C) (n = 2, 0.6%), and Hb St. Luke’s-Thailand (*HBA2*:c.287C>G) (n = 1, 0.3%), were identified in seven alleles (Table 1). The distribution of Hb variants among the southern population from ten provinces is shown in Fig. 1. The hematological parameters of 263 (78.0%) subjects with heterozygous Hb variant genotypes are shown in Table 2. Hb analysis showed that Hb Malay and Hb Dhonburi migrated along Hb A (zone 9). Furthermore, three Hb variants—HbTak, Hb Q-Thailand, and Hb G-Georgia—migrated to the Hb F zone (zone 7). Herein, we report Hb G-Georgia (*HBA1*) for the first time in Thailand (Fig. 2). Hb G-Georgia (*HBA1*) exhibited lower levels than Hb G-Georgia (*HBA2*), at 10.3–10.6% vs. 17.0%. Two Hb variants, Hb D-Punjab and Hb St. Luke ’s-Thailand, were migrated in zone 6. Notably, the levels of Hb D-Punjab in a case with compound heterozygous α^0^-thalassemia/α^+^-thalassemia (−−/−α) were lower than those in Hb D-Punjab carriers with the normal *HBA* gene (αα/αα) or heterozygous α^+^-thalassemia (−α/αα), at 23.6% vs. 32.6–39.3%. Moreover, Hb G-Makassar migrated to the Hb S (*HBB*:c.20A>T) zone (zone 5). Hb C, Hb Hope, Hb New York, and Hb J-Bangkok were detected in zones 2, 10, 11, and 12, respectively (Fig. 3).

**Table 1:** Mutation frequencies of hemoglobin (Hb) variants in a total of 346 chromosomes among 337 participants from each province of southern Thailand during 2015 to 2022.

Hemoglobin variant	No.	(%)
β-chain variants (9 mutations)		
Hb Malay	266	(76.9)
Hb C	35	(10.1)
Hb D-Punjab	10	(2.9)
Hb Dhonburi	8	(2.3)
Hb G-Makassar	8	(2.3)
Hb J-Bangkok	5	(1.4)
Hb Tak	5	(1.4)
Hb New York	1	(0.3)
Hb Hope	1	(0.3)
α-chain variants (4 mutations)		
Hb G-Georgia (*HBA1*)	3	(0.9)
Hb G-Georgia (*HBA2*)	1	(0.3)
Hb Q-Thailand	2	(0.6)
Hb St. Luke’s-Thailand	1	(0.3)

The number represents the allelic count of Hb variants.

**Table 2:** Hematological characteristics of 263 Hb variant carriers.

Hemoglobin variants	No. of cases	Gender (n)	Age range	α or β genotype	Hematological parameter					Hemoglobin analysis				
					Hb	Hct	MCV	MCH	RDW	Hb A_2_	Hb F	Hb variant	Hb variant zone	Hb pattern
			(years)		(g/dL)	(%)	(fL)	(pg)	(%)	(%)	(%)	(%)		
β-chain variants														
Hb Malay	178	F (84)	17–43	αα/αα	11.0 ± 0.9	34.3 ± 3.5	71.5 ± 4.0	23.1 ± 1.5	15.4 ± 1.1	4.5 ± 0.4	0.1 ± 0.7		9	A_2_A^a^
		M (94)	16–60		13.4 ± 1.1	41.9 ± 3.6	71.7 ± 3.5	23.0 ± 1.5	15.4 ± 1.9	4.5 ± 0.4	0.1 ± 0.6			
	20	F (10)	17–35	−α/αα	11.3 ± 1.3	35.0 ± 4.2	75.0 ± 2.5	24.3 ± 0.5	14.9 ± 0.8	4.6 ± 0.4	0			
		M (10)	18–41		13.7 ± 0.6	41.9 ± 1.6	76.3 ± 2.5	24.7 ± 0.7	14.6 ± 1.0	4.3 ± 0.3	0			
	3	F (1)	22	−−/αα	9.9	31.1	70	22.3	16.9	4.6	0			
		M (2)	21, 27		13.5, 11.5	39.3, 37.3	64.0, 70.0	21.8, 21.6	19.0, 15.4	4.3, 4.3	0			
Hb C	25	F (17)	3–75	αα/αα	11.8 ± 0.9	33.7 ± 4.0	78.2 ± 6.7	27.4 ± 1.9	14.0 ± 0.9	3.5 ± 0.2	0.9 ± 1.2	34.1 ± 1.6	2	A_2_A with Hb Var
		M (8)	1–65		14.5 ± 1.5	41.0 ± 5.0	79.0 ± 5.5	28.2 ± 2.2	13.6 ± 1.1	3.4 ± 0.3	2.0 ± 2.5	34.2 ± 1.3		
	1	F	33	−α/αα	11.1	35.1	65.5	20.7	NA	3.1	0	31.1		
Hb Dhonburi	8	F (4)	18–39	αα/αα	10.4 ± 1.4	32.3 ± 4.3	72.8 ± 4.2	22.9 ± 1.2	16.3 ± 1.9	4.2 ± 0.4	0		9	A_2_A^a^
		M (4)	22–36		13.7 ± 0.9	42.0 ± 3.4	76.5 ± 0.6	25.0 ± 0.4	13.2 ± 0.6	4.1 ± 0.3	0			
Hb D-Punjab	5	F (1)	26	αα/αα	11.0	34.0	81.1	26.3	12.4	3.2		39.3	6	A_2_A with Hb Var
		M (4)	15–55		11.1 ± 2.3	34.1 ± 4.8	68.5 ± 13.3	22.6 ± 5.8	15.8 ± 3.5	2.9 ± 0.3		35.2 ± 2.6		
	1	F	55	−α/αα	9.5	26.8	76.0	26.8	14.6	3.1		36.6		
	1	M	34	α^CS^α/αα	13.2	39.3	78.0	26.3	12.8	3.0		37.7, (CS = 0.5)	6, 2	A_2_ACS with Hb Var
	1	M	23	−−/−α	NA	NA	NA	NA	NA	3.2		23.6	6	A_2_A with Hb Var
Hb G-Makassar	5	F (2)	22, 56	αα/αα	12.1, 13.3	34.6, 37.3	76.0, 78.0	26.5, 27.7	13.8, 13.6	3.1, 3.3		43.5, 42.2	5	A_2_A with Hb Var
		M (3)	15–30		14.8 ± 0.3	43.7 ± 2.1	76.7 ± 2.1	27.2 ± 1.6	13.4 ± 1.2	3.4 ± 1.4		41.5 ± 0.9		
	1	F	22	−α/αα	10.8	34.5	63.0	19.7	NA	2.3		39.1		
Hb Tak	4	F (1)	35	αα/αα	11.7	35.2	83.0	27.7	18.2	3.5	31.4		7	A_2_F^b^A
		M (3)	22–39		17.8 ± 2.5	50.8 ± 6.1	82.3 ± 4.5	30.0 ± 1.9	17.7 ± 1.2	3.5 ± 0.2	32.2 ± 3.6			
Hb J-Bangkok	1	F	36	−α/αα	13.2	41.9	77.0	24.1	14.1	2.6	0.8	49.3	12	A_2_A with Hb Var
	1	M	57	−−/αα	7.7	25.4	70.0	24.4	18.0	2.3	0.4	48.4		
Hb New York	1	F	46	αα/αα	10.3	31.1	89.6	29.7	18.9	3		46.5	11	A_2_A with Hb Var
α-chain variants														
Hb G-Georgia (*HBA1*)	3	F (2)	18, 38	β/β	11.4, 12.0	36.1, 35.6	92.0, 82.0	29.0, 27.8	12.0, 12.6	2.3, 2.3	10.5, 10.3		7	A_2_F^b^A
		M (1)	40		13.9	41.3	88.0	29.4	13.1	2.8	10.6			
Hb G-Georgia (*HBA2*)	1	F	33	β^E^/β	NA	NA	72.0	NA	NA	3.6	17.0	(E = 20)	7, 4	EF^b^A
Hb Q-Thailand	1	M	38	β^CD41/42^/β	12.7	41.3	71.0	21.7	13.4	4.4	15.8		7	A_2_F^b^A
	1	F	33	β/β	11.7	36.2	79.0	25.5	13.3	1.9	28.6		7	
Hb St. Luke's-Thailand	1	M	29	β/β	16.8	49.5	94.0	31.8	11.3	1.8		18.9	6	A_2_A with Hb Var

^a^Hb variant co-migrated with Hb A, ^b^Hb variant co-migrated with Hb F, *NA* not available, *Hb Var* hemoglobin variant, *CS* Constant Spring, *F* female, *M* male, *CD* codon.

### Hematological profiles of patients with homozygous or compound heterozygous Hb variants

The hematological parameters of 61 (18.1%) patients with homozygous or compound heterozygous Hb variant genotypes without blood transfusion are shown in Table 3. The results showed that eight patients with homozygous Hb Malay and 27 patients with compound heterozygous Hb Malay with Hb E displayed mild-to-moderate anemia without a history of blood transfusion. Two patients with Hb Malay and β^+^-thalassemia (NT-28 (A>G), *HBB*:c.− 78A>G) exhibited nontransfusion-dependent thalassemia and moderate anemia. In addition, five cases of compound heterozygous Hb Malay with high Hb F determinant mutations, such as HPFH6 (NG_000007.3:g.45595_124872del), δβ^0^-thalassemia (12.5 kb deletion) (NG_000007.3:g.64383_76994del), Indian del-inv ^A^γδβ^0^-thalassemia (NG_000007.3:g.48400_49245del;49246_64567inv;64568_72051del), and Thai del-inv-ins ^A^γδβ^0^-thalassemia (NG_000007.3:g.47449_165744del;168412_168590invins;insAAGAAGA), along with one patient with compound heterozygous Hb Malay with β^0^-thalassemia (3.5 kb deletion, NC_000011.10:g.5224302-5227791del3490bp), exhibited a non-transfusion-dependent thalassemia phenotype. Among nine patients with compound heterozygous Hb C with other hemoglobinopathies, all were asymptomatic or presented mild anemia. This group included six patients with Hb C/Hb E, one patient with Hb C/Hb Malay, and two patients with Hb C/β^0^-thalassemia (3.5 kb deletion and codon 41 (− C), (*HBB*:c.126delC)). Interestingly, a patient with compound heterozygous Hb C and β^0^-thalassemia (3.5 kb deletion) displayed significantly elevated Hb A_2_ (7.6%) and Hb F (11.6%) levels. In contrast, a patient with compound heterozygous Hb C and β^0^-thalassemia (codon 41 (− C)) exhibited Hb A_2_ (3.7%) without detectable Hb F levels. This study reports several Hb variants co-inherited with Hb E in the southern population, including two cases of Hb D-Punjab/Hb E, two cases of Hb G-Makassar/Hb E, one case of Hb J-Bangkok/Hb E, and one case of Hb Hope/Hb E, all of which showed no clinical symptoms. Additionally, two patients with compound heterozygous Hb J-Bangkok with β^+^-thalassemia [IVS1-5 (G>C), (*HBB:*c.92 + 5G>C)] exhibiting mild anemia were reported for the first time. Hb analysis of the samples of these patients revealed Hb J-Bangkok in zone 12 (89.5% and 93.0%) and Hb A_2_ (> 3.5%). Finally, one patient with compound heterozygous Hb Tak and β^+^-thalassemia (IVS1-5 (G>C)) displayed 5.6% Hb A_2_ and 91.6% Hb Tak. However, this patient presented with mild anemia without secondary erythrocytosis.

**Table 3:** Hematological characteristics of 61 patients with homozygous or compound heterozygous Hb variants and hemoglobinopathies who were not dependent on transfusion.

Hemoglobin variants genotype	No. of cases	Gender (n)	Age range	α-genotype	Hematological parameter					Hemoglobin analysis					
					Hb	Hct	MCV	MCH	RDW	Hb A_2_	Hb E	Hb F	Hb variant	Hb variant	Hb pattern
			(years)		(g/dL)	(%)	(fL)	(pg)	(%)	(%)	(%)	(%)	(%)	zone	
Homozygous genotype															
Hb Malay/Hb Malay	8	F (2)	9, 14	αα/αα	8.7, 8.7	27.0, 28.8	50.3, 51.7	16.2, 15.6	29.8, 26.5	5.4, 4.5		42.7, 45.3		9	A_2_FA^a^
		M (6)	20–33		10.0 ± 1.7	30.6 ± 5.1	58.2 ± 5.7	19.7 ± 0.7	24.1 ± 0.9	5.2 ± 1.0		37.6 ± 9.8			
Compound heterozygous genotype															
Hb Malay/Hb E	27	F (12)	2–35	αα/αα	9.1 ± 0.8	27.8 ± 2.0	61.7 ± 12.1	18.6 ± 1.4	21.0 ± 2.0	5.4 ± 0.5	48.5 ± 4.2	10.9 ± 4.6		9, 4	EFA^a^
		M (15)	2–37		9.7 ± 1.4	30.4 ± 4.6	54.3 ± 5.1	17.4 ± 1.3	20.7 ± 2.3	5.8 ± 0.7	47.7 ± 5.3	8.2 ± 7.8			
Hb Malay/β^+^-thal (NT-28 (T > C))	1	F	13	αα/αα	7.7	23.7	63.0	20.5	26.3	NA		NA	NA	NA	NA
	1	F	1	–α/αα	9.1	28.9	58.3	18.3	27.9	NA		NA	NA	NA	NA
Hb Malay/Hb C	1	F	1	αα/αα	10.3	31.4	58.0	19.0	14.8	4.6		8.3	65.7	9, 2	A_2_A^a^ with Hb Var
Hb Malay/δβ^0^-thal (12.5 kb deletion)	3	F (2)	2–44	αα/αα	9.4, 10.0	28.0, 29.8	68.0, 61.1	21.0, 20.6	20.5, 22.4	2.6, 2.3		70.0, 72.9		9	A_2_FA^a^
		M (1)			10.5	33.4	68.0	21.3	20.7	2.4		61.4			
Hb Malay/Indian del-inv ^A^γδβ^0^-thal	1	F	11	αα/αα	9.7	30	55	17.8	25.7	3.1		62.2		9	A_2_FA^a^
Hb Malay/Thai del-inv-ins ^A^γδβ^0^-thal	1	M	29	αα/αα	13.7	43.0	63.0	20.0	22.1	2.6		68.0		9	A_2_FA^a^
Hb Malay/β^0^-thal (3.5 kb deletion)	1	F	38	––/αα	9.1	27.3	61.9	20.6	27.2	8.7		52.6		9	A_2_FA^a^
Hb C/Hb E	4	F (2)	2–79	αα/αα	11.1, 10.8	30.8, 30.2	62.7, 65.0	22.6, 23.2	15.3, 16.2	4.1, 4.0	30.6, 32.0	3.1, 3.5	54.7, 56.0	2, 4	E with Hb Var
		M (2)			11.7, 15.0	31.8, 45.0	59.0, 74.0	21.7, 25.1	17.2, 16.4	3.6, 4.1	33.0, 34.0	4.0, 2.0	54.9, 53.7		
	1	M	53	α^CS^α/αα	9.5	26.7	71.0	25.3	15.2	4.2	34.7	5.5	55.3	2, 4	ECS with Hb Var
	1	M	22	––/αα	12.6	35.8	57.0	19.9	18.0	4.4	35.6	1.7	58.3	2, 4	E with Hb Var
Hb C/β^0^-thal (3.5 kb deletion)	1	F	60	αα/αα	10.7	32.3	53.5	17.7	22.9	7.6		11.6	79.2	2	A_2_F with Hb Var
Hb C/β^0^-thal (codon41 (− C))	1	M	24	αα/αα	12.4	37.7	58.9	19.4	20.0	3.7		0	91.2	2	A_2_ with Hb Var
Hb D-Punjab/Hb E	2	F, M	28, 29	–α/αα	15.1, 12.0	44, 33.2	79.0, 69.0	27.0, 24.9	14.0, 15.6	3.8, 3.7	24.5, 29.0	5.1,0.0	66.6, 65.6	6, 4	E with Hb Var
Hb G-Makassar/Hb E	2	M	38, 74	αα/αα	15.7, 12.3	44.6, 35.4	71.0, 64.7	24.9, 22.5	14.1, 15.5	5.2, 4.9	23.3, 24.8		69.2, 68.8	5	E with Hb Var
Hb J-Bangkok/β^+^-thal (IVS1-5 (G>C))	2	M, F	30, 56	αα/αα	10.1, 10.6	33.6, 35.6	62.0, 67.9	18.6, 20.2	17.5, 15.0	4.8, 4.2		2.6, 0.0	89.5, 93.0	12	A_2_A with Hb Var
Hb J-Bangkok/Hb E	1	F	39	αα/αα	11.0	33.6	72.0	23.6	13.1	3.9	23.2		72.0	12, 4	E with Hb Var
Hb Tak/β^+^-thal (IVS1-5 (G>C))	1	F	4	αα/αα	11.8	37.5	69.7	21.9	29.5	5.6		91.6		7	A_2_F^b^
Hb Hope/Hb E	1	F	47	αα/αα	11.0	34.4	80.4	25.7	14.1	3.4	22.0		72.7	10, 4	E with Hb Var

^a^Hb variant co-migrated with Hb A, ^b^Hb variant co-migrated with Hb F, *NA* not available, *Hb Var* hemoglobin variant, *thal* thalassemia, *F* female, *M* male, *NT* nucleotide, *IVS* intervening sequence.

Furthermore, 13 patients with compound heterozygous Hb Malay, harboring various β-thalassemia point mutations [e.g., NT-28 (A>G), codon 17 (A>T) (*HBB*:c.52A>T), codon 41 (− C), codons 41/42 (− TTCT) (*HBB*:c.126_129delCTTT), IVS1-1 (G>T) (*HBB*:c.92 + 1G>T), IVS1-5 (G>C), and IVS2-654 (C>T) (*HBB*:c.316-197C>T)], were identified as transfusion-dependent thalassemia cases who need regular blood transfusion to manage their clinical complications and survival. The frequency of blood transfusion, clinical history, and hematological profiles in these patients is shown in Table 4.

**Table 4:** Hematological characteristics of 13 patients with compound heterozygous Hb variants and hemoglobinopathies who were dependent on transfusion.

Hemoglobin variants genotype	No. of cases	Gender (n)	Age	Frequency of blood transfusion	Splenectomy	Underlying condition	α-genotype	Hematological parameter after regular blood transfusion				
			(years)		(yes/no)			Hb	Hct	MCV	MCH	RDW
								(g/dL)	(%)	(fL)	(pg)	(%)
Hb Malay/β^+^-thal (NT-28 (T>C))	1	M	13,	Every two months	No	Splenomegaly	αα/αα	5.3,	19.1	80.0	22.1	27.5,
Hb Malay/β^0^-thal (codon 17 (A>T))	2	M, F	10, 33	Every two months	No, yes	NA, Secondary hemochromatosis and secondary pulmonary hypertension	αα/αα	7.6, 4.4	23.3, 16.6	78.0, 73.0	25.3, 22.0	23.4, 25.6
Hb Malay/β^0^-thal (codon 41 (-C))	1	M	24	Once a month	Yes	Secondary hemochromatosis	αα/αα	6.8	20.2	77.0	26.1	18.7
Hb Malay/β^0^-thal (codons 41/42 (-TTCT))	3	F (2)	2, 14	Once a month, every two months	No	NA	αα/αα	4.4, 8.7	13.9, 27.2	52.7, 72.0	16.7, 22.5	33.0, 20.9
		M (1)	29	Once a month	No	Hepatosplenomegaly		NA	NA	NA	NA	NA
Hb Malay/β^0^-thal (IVS1-1 (G>T))	2	M	1, 3	Once or twice a month	No	NA	αα/αα	7.3, 6.0	22.2, 19.6	66.0, 65.0	21.6, 19.8	35.4, 26.5
Hb Malay/β^+^-thal (IVS1-5 (G>C))	3	F (1)	11	Every two months	Yes	NA	αα/αα	6.5	23.0	64.0	18.6	24.4
		M (2)	24, 26	Once a month, twice a month	No	NA, Chronic renal disease		6.5, 5.7	21.9, 17.9	73.0, 65.0	25.5, 26.5	22.1, 17.9
Hb Malay/ β^+^-thal (IVS2-654 (C>T))	1	M	2	twice a month	No	NA	αα/αα	7.5	23.0	64.0	20.8	30.1

*NA* not available, *thal* thalassemia, *F* female, *M* male, *NT* nucleotide, *IVS* intervening sequence.

Table 5 displays a comparative analysis of the spectrum of Hb variants in the population of southern Thailand and the populations from other areas. The findings indicate distinct prevalence patterns of Hb variants across various parts of the country. Hb Malay and Hb C were the predominant variants in the southern population, whereas Hb Hope, Hb Q-Thailand, and Hb J-Bangkok were frequently found in populations from the northern and central regions. The northeastern population presented the prevalence of four common mutations—Hb Q-Thailand, Hb J-Bangkok, Hb Pyrgos (*HBB*:c.251G>A), and Hb Hope. Moreover, northeastern Thailand populations displayed notable variability in the distribution of Hb variants.

**Table 5:** Comparison of the molecular spectrum of Hb variants among 337 participants from southern Thailand and that reported in previous studies in populations from different parts of the country.

Hemoglobin variants	HGVS nomenclature	South	South	South	Center	Northeast	North
		This study	^7^	^1^	^1^	^1^	^2^
		(%)	(%)	(%)	(%)	(%)	(%)
β-chain variants							
Hb Malay	*HBB*:c.59A>G	**76.6**	ND	**29.3**	3.3	4.6	ND
Hb C	*HBB*:c.19G>A	**10.1**	**50.0**	**17.4**	3.0		0.5
Hb D-Punjab	*HBB*:c.364G>C	3.0	**21.4**	**23.9**	4.1	2.3	
Hb G-Makassar	*HBB*:c.20A>C	2.4	7.1				0.5
Hb Dhonburi	*HBB*:c.380T>G	2.4				3.7	ND
Hb J-Bangkok	*HBB*:c.170G>A	1.5		1.1	**8.6**	8.7	1.5
Hb Tak	*HBB*:c.441_442insAC	1.5	12.5	13.0	**14.5**	**16.9**	**14.6**
Hb New York	*HBB*:c.341T>A	0.3					
Hb Hope	*HBB*:c.410G>A	0.3	1.8	2.2	**32.0**	**12.3**	**55.8**
Hb S	*HBB*:c.20A>T			1.1			1.5
Hb Korle-Bu	*HBB*:c.220G>A			1.1	3.3	4.1	0.5
Hb Pyrgos	*HBB*:c.251G>A				5.2	**12.8**	
Hb Cook	*HBB*:c.398A>C				0.7	3.7	
Hb Dhofar	*HBB*:c.176C>G				0.7		
Hb J-Kaohsiung	*HBB*:c.179A>C					1.4	
Hb Phimai	*HBB*:c.218G>C					0.9	
Hb Raleigh	*HBB*:c.5T>C					0.5	
Hb Khon Kaen	*HBB*:c.370_378delACCCCACCA						
Hb E-Saskatoon	*HBB*:c.67G>A					0.5	
α-chain variants							
Hb G-Georgia (*HBA1*)	*HBA1*:c.287C>T	0.9					
Hb G-Georgia (*HBA2*)	*HBA2*:c.287C>T	0.3					
Hb Q-Thailand	*HBA1*:c.223G>C	0.6	3.6	5.4	**20.4**	**18.3**	**19.4**
Hb St. Luke’s-Thailand	*HBA2*:c.287C>G	0.3				0.9	
Hb O-Indonesia	*HBA1*:c.349G>A		3.6				
Hb Queens	*HBA1*:c.104T>G			5.4	0.4		
Hb Siam	*HBA2*:c.46G>C(or *HBA1*)				1.9	1.4	1.0
Hb Beijing	*HBA2*:c.51G>C(or *HBA1*)				0.4		0.5
Hb Nakhon Ratchasima	*HBA2*:c.191C>T				0.7	1.4	
Hb G-Honolulu	*HBA2*:c.91G>C(or *HBA1*)				0.4		
Hb J-Wenchang-Wuming	*HBA2*:c.34A>C(or *HBA1*)				0.4		
Hb Hekinan	*HBA1*:c.84G>C					2.3	3.9
Hb Dunn	*HBA2*:c.19G>A(or *HBA1*)					1.4	
Hb Thailand	*HBA1*:c.170A>C					1.4	
Hb Q-India	*HBA1*:c.193G>C					0.5	
Hb Phnom Penh	*HBA1*:c.354_355insATC					0.5	
Hb Kawachi	*HBA2*:c.134C>G(or *HBA1*)						0.5
Total cases		337	56	92	269	219	206

The common Hb variants in each part are presented in bold. *ND* not done.

## Discussion

**Figure 4:**
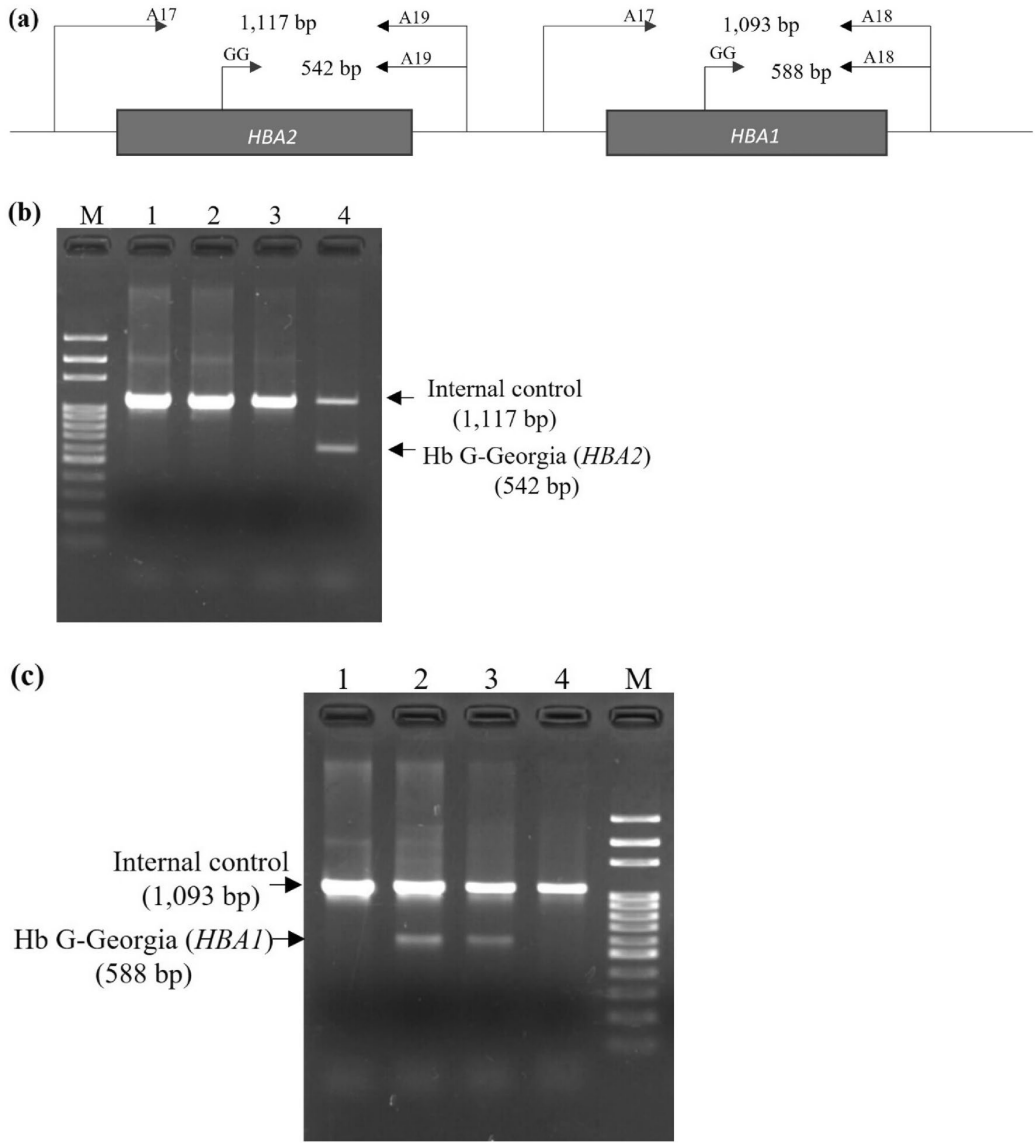
Schematic illustrating the primer orientation for the newly developed allele-specific PCR (**a**). Agarose gel electrophoresis results for the detection of hemoglobin (Hb) G-Georgia (*HBA2*) (**b**) and Hb G-Georgia (*HBA1*) (**c**). M; 100 bp DNA marker, 1; negative for Hb G-Georgia (*HBA2* and *HBA1*), 2 and 3; positive for Hb G-Georgia (*HBA1*), 4; positive for Hb G-Georgia (*HBA2*).

This study revisited the molecular spectrum of Hb variants in the southern population across ten provinces through a large-scale survey of specimens received by our center over eight years. Among 13,391 individuals, 337 (2.5%) carried Hb variants, with 263 (78%) identified as Hb variant carriers. The predominant Hb variant was Hb Malay, followed by Hb C. Herein, we report rare Hb variants identified in zone 7, similar to Hb F, including Hb Q-Thailand, Hb Tak, and Hb G-Georgia. To our knowledge, this study is the first to report Hb G-Georgia on the *HBA1* gene in Thailand. Hb G-Georgia (*HBA1*) heterozygote showed lower levels (10.3–10.6%) than Hb G-Georgia (*HBA2*), 17.0% in a double heterozygote Hb G-Georgia and Hb E in this study and 23.4% in Hb G-Georgia heterozygote reported in a previous study^8^, which may be explained by approximately 2–3 times lower expression of *HBA1* gene than the *HBA2* gene^9^. Interestingly, Hb G-Georgia did not present any Hb A_2_ variant peak in hemoglobin analysis using the CE technique, observed in both heterozygote Hb G-Georgia and double heterozygote Hb G-Georgia and Hb E. Thus, this could be misconstrued as β^+^/β^+^ or β^+^/β^0^-thalassemia disease or β^+^-thalassemia with Hb E disease based on the Hb pattern, reflecting A_2_FA or EFA. However, the three patients with heterozygous Hb G-Georgia in our study exhibited no clinical symptoms and normal RDW levels. Furthermore, an alkaline denaturation test yielded negative results. Therefore, we performed DNA sequencing to identify this Hb variant. A patient harboring compound heterozygous Hb G-Georgia with α^0^-thalassemia did not develop Hb H disease^4^, indicating that Hb G-Georgia is not classified as an α-thalassemia mutation. However, rapid molecular diagnosis is required for proper genetic counseling. Thus, we developed an allele-specific PCR (AS-PCR) for detecting Hb G-Georgia in both *HBA1* and *HBA2* genes for the first time (Fig. 4). Unlike PCR–RFLP, this technique is simple, rapid, inexpensive, and does not require restriction enzymes^4^.

The clinical phenotype of Hb Tak often includes erythrocytosis in patients with compound heterozygous Hb Tak with β-thalassemia, homozygous Hb Tak, and Hb Tak with δβ^0^-thalassemia^10–12^. However, a patient with Hb Tak and β^+^-thalassemia (IVS1-5 (G>C)) showed no symptomatic erythrocytosis (Hb 11 g/dL, Hct 37.5%), which might be explained by the underlying disease with an atrial septal defect and failure to thrive.

Thalassemia mutations are common and heterogeneous in southern populations^13^. We reported the interaction of Hb Malay with other abnormalities resulted in diverse genotypes in 57 (16.9%) patients. The most common genotype was compound heterozygous Hb Malay with Hb E patients (n = 27), manifesting a thalassemia intermedia phenotype without blood transfusion, similar to those reported previously^14,15^. Accordingly, prenatal diagnosis is deemed unnecessary for couples at risk of developing Hb Malay with Hb E disease to reduce the risk of miscarriage. Nevertheless, postnatal diagnosis and appropriate genetic counseling are imperative. This study showed that Hb Malay with β^0^-thalassemia, including codon 17 (A>T), codon 41 (− C), codons 41/42 (− TTCT), IVS1-1 (G>T), or β^+^-thalassemia, including IVS1-5 (G>C) and IVS2-654 (C>T), led to severe anemia, wherein patients required regular blood transfusion. Prenatal diagnosis is thus essential for families with this combination. Conversely, a patient with Hb Malay and β^0^-thalassemia (3.5 kb deletion) presented with moderate anemia (Hb 9.1 g/dL) without the need for blood transfusion. This milder clinical manifestation could be due to co-inheritance with heterozygous α^0^-thalassemia, ameliorating clinical severity by balancing the levels of α- and β-globin chains^16–19^. Accordingly, this study supports a previous recommendation proposing the inclusion of α^0^-thalassemia analysis in prenatal diagnosis for fetuses affected with thalassemia disease to make appropriate decisions^20^. Previous studies reported that β^0^-thalassemia (3.5 kb deletion) carriers usually exhibit higher Hb A_2_ and Hb F levels than other β-thalassemia carriers due to point mutations^21,22^. The positive result in reverse dot blot (RDB) hybridization indicating a homozygous Hb Malay genotype in the Hb Malay with β^0^-thalassemia (3.5 kb deletion) case, alongside hematological profiles resembling thalassemia intermedia, raises the possibility of misdiagnosis as homozygous Hb Malay. However, the elevated Hb A_2_ levels (8.7%) compared to those of homozygous Hb Malay (4.5–5.4%) underscored the need for further laboratory investigation into β^0^-thalassemia (3.5 kb deletion). Subsequently, the true genotype of this patient was found to be Hb Malay with β^0^-thalassemia (3.5 kb deletion).

Interestingly, a previous study reported that Hb Malay with β^+^-thalassemia typically manifests as a thalassemia intermedia phenotype without the need for regular blood transfusion^15^. However, this study reported three cases of Hb Malay with β^+^-thalassemia (NT-28 (A>G)) exhibiting distinct phenotypes. One patient presented transfusion-dependent thalassemia and splenomegaly; this was potentially influenced by additional abnormalities. Subsequently, gap-PCR was conducted to identify α-globin gene triplication (ααα/αα)^23^, which, if present, could exacerbate globin chain imbalance and escalate clinical severity^24^. Despite obtaining a negative result for this patient (data not shown), we propose the application of whole-exome sequencing to comprehensively determine the clinical severity.

In Thailand, the frequency of high Hb F determinants is 1.06%^25^. The co-occurrence of this abnormality with β-thalassemia can yield diverse clinical phenotypes, ranging from mild to severe anemia, depending on the β-thalassemia genotype^25–27^. However, scant information exists on the clinical phenotypes associated with high Hb F determinants in patients with Hb Malay. We present, for the first time, a case of Hb Malay with Thai del-inv-ins ^A^γδβ^0^-thalassemia exhibiting no clinical symptoms. Moreover, combinations of Hb Malay with δβ^0^-thalassemia (12.5 kb deletion), Indian del-inv ^A^γδβ^0^-thalassemia, or HPFH6 also presented only thalassemia intermedia phenotype without the need for blood transfusion. These results suggest that prenatal diagnosis might be unnecessary for couples carrying Hb Malay with high Hb F determinants. However, a postnatal diagnosis should be performed for proper genetic counseling.

Hb J-Bangkok is a β-chain variant occasionally reported in Thailand. A carrier usually presents with normal hematological parameters, with Hb J-Bangkok levels of 44.5 ± 4.7%^28^. However, we report a case of Hb J-Bangkok carrier with moderate anemia (Hb 7.7 g/dL), potentially affected by an underlying disease but unconfirmed patient-specific condition. Interestingly, we report two cases of Hb J-Bangkok with β^+^-thalassemia (IVS1-5 (G>C) for the first time. Elevated Hb J-Bangkok levels of 89.5% and 93% in patients with mild anemia (Hb 10.1 and 10.6 g/dL) might suggest the presence of homozygous Hb J-Bangkok. However, this rare variant is infrequently reported in southern populations. Thus, these patients are preferably linked to co-inheritance with β-thalassemia mutation, and molecular diagnosis of β-thalassemia is subsequently performed in these cases.

The levels of Hb E or Hb C in pure Hb E or pure Hb C heterozygotes were higher than those genotypes co-inherited with α^0^-thalassemia because the α-globin chain prefers to form dimerization with β-globin chain than β^E^ or β^C^-globin chain. Thus, reduced α-globin chain production in α^0^-thalassemia contributes to lower Hb E or Hb C levels. However, Hb E levels in compound heterozygous Hb C/Hb E are higher than in Hb E heterozygote. The previous studies reported that the Hb E levels in compound heterozygous Hb C/Hb E could be presented in a wide range from 32.0 to 39.7%^14,29^. This study reported four cases with compound heterozygous Hb C/Hb E without co-inherited α-thalassemia with Hb E levels ranging from 30.6 to 34.0%, while a compound heterozygous Hb C/Hb E co-inherited α^0^-thalassemia presented with Hb E levels of 35.6%. Hence, lower levels of Hb E were not observed in a compound heterozygous Hb C/Hb E co-inherited with α^0^-thalassemia. It might be due to both Hb E and Hb C are positively charged Hb variants, which might have a similar ability to interact with the α-globin chain. Moreover, the decrease of αβ^C^ dimer formation leading to an indirect increase in the αβ^E^ dimer formation^29^ as the same as that presented in compound heterozygous Hb S/Hb E disease^30^.

For two cases of compound heterozygous Hb D-Punjab/Hb E co-inherited with α^+^-thalassemia, the Hb D-Punjab value (66.6% and 65.6%) is elevated while the Hb E value (24.5% and 29.0%) is the same as the Hb E heterozygote. Hb D-Punjab mutation results in structural protein changes but does not affect the value of Hb D-Punjab production. However, Hb E mutation creates abnormal mRNA splicing, resulting in low output of Hb E. Thus, lower Hb E levels than Hb D-Punjab levels could be observed in the compound heterozygous Hb D-Punjab/Hb E cases. Moreover, co-inherited α^+^-thalassemia in compound heterozygous Hb D-Punjab/Hb E cases might not much affect the lower production of Hb E levels when compared to Hb E levels of compound heterozygous Hb D-Punjab/Hb E with normal α-globin chain cases in a previous report (24.5% and 29.0% vs 28.4% and 29.3%)^31^.

The Hb variant spectra in Thailand were compared. Three common Hb variants—Hb Hope, Hb Q-Thailand, and Hb Tak—have been observed in many populations from northern, central, and northeastern Thailand^1^. However, the southern population showed different common Hb variants, especially compared to the northern population. It could be explained by differing ethnic backgrounds of populations between the north and south. In Thailand, most people belong to the Thai ethnicity. However, each part of the country has different minority ethnic groups. Minor ethnic groups were observed in the northern population, including Lawa, Mon, Shan, Yuan, Khuen, Lue, and Yong^32^. By contrast, the minority ethnic groups in the southern population are Thai Muslims, Maniq, Moken, Moklen, and Urak Lawoi^33^. Furthermore, the mitochondrial phylogenetic analysis revealed that the population from the northern area has distinct haplotype groups compared to those of the southern population^34^. This divergence may be explained by the proximity of southern Thailand to the sea, leading to populations of diverse nationalities due to human migration from neighboring countries such as Malaysia and India, where Hb Malay and Hb D-Punjab are prevalent, respectively^35,36^. In addition, Hb C is commonly found in West African populations^37^ and is occasionally reported in Southeast Asian populations of different origins^29^. Carriers of Hb C are immune to malarial infections^38^. Accordingly, Hb C is predominantly observed in southern Thailand, where malaria is endemic. Moreover, Hb G-Makassar is frequently observed in the southern population, similar to that in the Malaysian population^39^. Hb G-Makassar comigrated at the same retention time as that for Hb S, as determined using the CE technique. Thus, molecular testing is essential for differential diagnosis. Furthermore, we reported two cases of Hb G-Makassar with Hb E, presenting mild clinical phenotypes similar to that in a previous report^39^.

In conclusion, this study demonstrates a distinct spectrum of Hb variants in Thailand. In addition, we describe the clinical aspects of Hb variants in combination with thalassemia or hemoglobinopathies. This information is essential for determining the need to perform prenatal diagnosis in the prevention and control program for thalassemia in this region.

## Materials and methods

All laboratory methods were performed following the national guidelines of Thailand for laboratory diagnosis of thalassemia and hemoglobinopathy^40^. The study protocol was approved by the Human Research Ethics Unit (HREU) of the Faculty of Medicine, Prince of Songkla University (REC 63-458-5-2). Consent was obtained from all 337 participants with Hb variants. Participants who visited or had their blood samples collected were referred to Songklanagarind Hospital from 2015 to 2020. Informed consent was obtained via telephone, followed by sending the documents via the post office. For patients who visited Songklanagarind Hospital from 2021 to 2022, informed consent was obtained when they visited for follow-up.

Each hospital provided hematological profiles and recorded the history of blood transfusion data. The hematological profiles and history of blood transfusion data of the patients from ten provinces were collected from the laboratory requesting program of Songklanagarind Hospital. We collected hematological data, history of blood transfusion, routine molecular diagnosis results, and DNA samples of patients referred to Songklanagarind Hospital from January 2015 to December 2022 for diagnosing thalassemia.

### Samples

A total of 337 DNA specimens were obtained from molecular diagnosis at the thalassemia unit at the Department of Pathology, Faculty of Medicine, Prince of Songkla University, southern Thailand.

### Hematological analysis

The hematological profiles were obtained from each hospital in the ten provinces. In our center, hematological parameters were obtained from an automated blood cell counter (Sysmex XN 3000; Sysmex, Japan). Hemoglobin analysis of all referred samples was performed using CE technique (Capillarys 2; Sebia, Lisses, France) at our laboratory, and a thorough review of blood transfusion history was conducted.

### Molecular analysis

Routine molecular diagnosis was performed to identify β-thalassemia, α-thalassemia, Hb Hb CS, Hb Paksé (PS, *HBA2*:c.429A>T), and high Hb F determinants using PCR-based techniques. Analysis of point mutations in β-thalassemia involved the examination of Hb Malay, Hb Dhonburi, and β-thalassemia 19 common mutations in southern Thailand were performed using RDB hybridization^13^. Analysis of β-thalassemia deletion (3.5 kb and 45 kb deletion (NG_000007.3:g.66258_184734del118477)) was carried out through melt-curve analysis^41^. High Hb F determinants, including δβ^0^-thalassemia (12.5 kb deletion), Indian del-inv ^A^γδβ^0^-thalassemia, HPFH6, and Thai del-inv-ins ^A^γδβ^0^-thalassemia (or Siriraj deletion), were identified using multiplex gap-PCR^42^. Nine α-thalassemia deletion mutations, such as −−^SEA^ (NC_000016.10:g.165397_184700), −−^THAI^ (NC_000016.10:g.149863_183312), −−^SA^ (NG_000006.1:g.19464_43064del23601), −−^CR^ (NC_000016.10:g.144,215_188,841), −−^FIL^ (NG_000006.1:g.11684_43534del31851), −−^MED^ (NG_000006.1:g.24664_41064del16401), -(α)^20.5^, -α^3.7^ (NG_000006.1:g.34164_37967del3804) and -α^4.2^ ((NC_000016.10:g.149863_183312), were identified using multiplex gap-PCR^43^. Allele-specific PCR was performed to identify Hb CS and Hb PS^44^. Additional investigation for unidentified Hb variants in the* HBA *and* HBB* genes among cases with Hb variant peak by CE method was confirmed by Sanger DNA sequencing using an ABI PRISMTM 3130xl analyzer (Applied Biosystems, Foster City, CA, USA) or by performing barcode-tagged sequencing based on next-generation sequencing on the Illumina MiSeq (Illumina, Inc., San Diego, CA).

### Development of allele-specific PCR for identification of Hb G-Georgia in the *HBA1 *and *HBA2* genes

Two AS-PCR conditions were developed for the differential diagnosis of Hb G-Georgia. To identify Hb G-Georgia in *HBA1*, a 1093 bp fragment generated from primers A17 (5′-GCTCCGCGCCAGCCAATGAG-3′) and A18 (5′-CTGGACTTCGCGGTGGCTC-3′) was used as an internal control. A 588 bp fragment specific to Hb G-Georgia in *HBA1* was amplified using primer GG (5′-ACAAGCTTCGGGTGGACCT-3′) and primer A18. For the identification of Hb G-Georgia in *HBA2*, a 1,117 bp fragment generated from primers A17 and A19 (5′-GCAGGCCTGGCACCTCTCAG-3′) was used as an internal control. A 542 bp fragment specific to Hb G-Georgia in *HBA2* was amplified using primers GG and A19 (Fig. 4). Each PCR reaction (25 μL) comprised 50–200 ng genomic DNA, 0.32 pmol of primers A17 and GG, 0.48 pmol of primers A18 or A19, 200 μM dNTPs, 1 M Betaine, 1.75 mM MgCl_2_, 1.25% DMSO, and 0.5 units of *Taq* DNA polymerase (Vivantis Technologies, Selangor Darul Ehsan, Malaysia) in 16 mM (NH_4_)_2_SO_4_ and 50 mM Tris–HCl (pH 9.2) buffer, and 0.1% Triton™ X-100. The cycling conditions were as follows: initial denaturation at 95 °C for 5 min, followed by 30 cycles at 94 °C for 1 min, 65 °C for 45 s, and 72 °C for 1 min 20 s, with a final extension at 72 °C for 5 min on a SimpliAmp™ thermal cycler (Thermo Fisher Scientific, Waltham, MA, USA). PCR products were separated via 1.5% agarose gel electrophoresis for 30 min. The amplified fragments were detected under UV light after staining with ethidium bromide (Supplmentary Figure S1).

### Supplementary Information

Supplementary Figure S1.

## Data Availability

The datasets generated and/or analyzed in the current study are available from the corresponding author upon reasonable request.
