# Supplementary material for: Molecular epidemiology and hematological profiles of hemoglobin variants in southern Thailand
Source: Sci Rep. 2024 Apr 22;14:9255. doi: 10.1038/s41598-024-59987-4 (PMC11035545; doi:10.1038/s41598-024-59987-4)

Supplementary Figure 1. Agarose gel electrophoresis results for the detection of hemoglobin (Hb) G-Georgia (*HBA2*) (right) and Hb G-Georgia (*HBA1*) (left). M; 100 bp DNA marker, 1; negative for Hb G-Georgia (*HBA2* and *HBA1*), 2 and 3; positive for Hb G-Georgia (*HBA1*), 4; positive for Hb G-Georgia (*HBA2*)

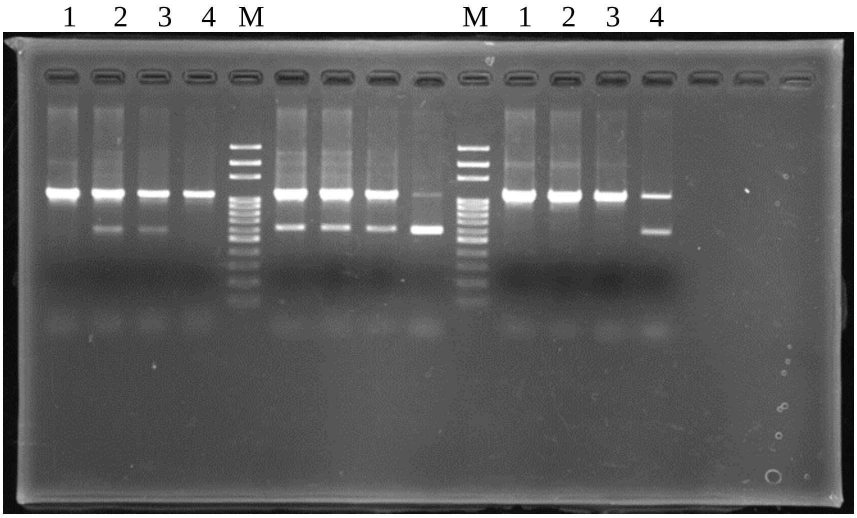

Supplement: Supplementary file 1 — Supplementary Figure S1. [file 41598_2024_59987_MOESM1_ESM.pdf]
